# Machine learning predicts clinical response to platelet-rich plasma therapy in knee osteoarthritis

**DOI:** 10.3389/fmed.2026.1787872

**Published:** 2026-04-14

**Authors:** Haokang Zhang, Xinhua Yang, Tianxiang Geng, Ruofei Wang, Guishi Li

**Affiliations:** 1 Qingdao University, Qingdao, Shandong, China; 2 Department of Orthopaedics, The Affiliated Yantai Yuhuangding Hospital of Qingdao University, Yantai, Shandong, China

**Keywords:** Gradient Boosting Classifier, knee osteoarthritis, machine learning, personalized therapy, platelet-rich plasma, SHAP analysis

## Abstract

**Objective:**

The purpose of the study is to predict clinical responses to platelet-rich plasma (PRP) therapy in patients with knee osteoarthritis (KOA) before treatment and to identify the crucial efficacy predictors.

**Methods:**

We reviewed the multidimensional clinical data of 102 KOA patients who underwent PRP therapy with a retrospective approach, including anthropometrics and blood indices. We defined the response to treatment as a reduction in the Numerical Rating Scale (NRS) pain score of ≥2 at the 6-month follow-up. After comprehensive data preprocessing, including imputation, Yeo–Johnson transformation, standardization, and balancing via SMOTETomek, 33 clinical features were retained. We then created and verified multiple machine learning models using 10-fold cross-validation (CV) on a 70% training cohort. The most effective model was subsequently validated on the 30% test cohort, and feature contributions were interpreted using SHapley Additive exPlanations (SHAP) analysis.

**Results:**

The Gradient Boosting Classifier showed the best overall performance among all tested models. The final model achieved a prediction accuracy of 90.3%, an area under the curve (AUC) of 0.862, and a Cohen’s kappa coefficient of 0.611 on the test set, indicating high predictive consistency. The SHAP interpretability analysis revealed three biomarkers—osmotic pressure, lipoprotein(a) [Lp(a)], and uric acid—as the clinical factors most strongly associated with treatment response.

**Conclusion:**

Clinical character-based machine learning models are effective in predicting PRP therapy outcomes prior to treatment. Such results provide a strong basis for introducing individualized therapeutic modalities in the management of osteoarthritis.

## Introduction

Knee osteoarthritis (KOA) is a chronic, progressive joint disorder characterized by articular cartilage degeneration, osteophyte formation, and subchondral bone remodeling, ultimately presenting clinically with debilitating pain and functional impairment (1–3). In contemporary orthopedic practice, intra-articular platelet-rich plasma (PRP) injection therapy has emerged as an increasingly adopted regenerative modality, particularly for patients who are refractory to conservative management, including physical therapy and oral analgesics (4–7). Mechanistically, PRP is theorized to promote tissue repair and modulate local inflammatory responses through the supratherapeutic concentration and release of autologous growth factors (7, 8). However, despite the widespread utilization of PRP therapy, empirical clinical observations indicate significant inter-individual heterogeneity in treatment response (9–12). Treatment failure not only delays effective interventions but also imposes substantial physical, temporal, and economic burdens on patients (13). Consequently, accurately predicting a patient’s clinical responsiveness to PRP prior to treatment has become an urgent therapeutic challenge (14).

Identifying robust preoperative predictors is fundamental for reliable patient stratification and the implementation of precision medicine (15, 16). Historically, prognostic research has predominantly relied on isolated clinical or demographic variables—such as age, sex, body mass index (BMI), or radiographic severity (15, 17).

Nevertheless, these unidimensional or narrowly focused indicators often fail to capture the systemic metabolic and physiological complexities underlying treatment efficacy, resulting in critically suboptimal predictive performance (18–20). Furthermore, the majority of prior studies have utilized conventional linear statistical models, which are inherently limited in revealing complex, non-linear interactions obscured within high-dimensional clinical datasets. Therefore, traditional methodologies remain insufficient to accurately stratify individual patients or to mechanistically explain the pronounced variance in PRP treatment outcomes (21, 22).

In recent years, advancements in machine learning (ML) architectures have provided paradigm-shifting solutions to these prognostic limitations (4, 18). In contrast to classical statistics, modern ML algorithms excel at processing high-dimensional data, autonomously discovering complex patterns within intricate clinical and serological profiles to optimize prediction accuracy (23–25). To overcome the inherent “black-box” limitations of advanced ML algorithms, novel model-agnostic interpretability frameworks, such as SHapley Additive exPlanations (SHAP), enable researchers to quantitatively map the precise marginal contribution of each input feature to the final prediction (4, 18, 23). By merging high-precision ML with rigorous *post-hoc* interpretability, this approach not only predicts outcomes but also reveals the biological networks and key clinical factors driving therapeutic efficacy (26).

In this study, we comprehensively collected multi-dimensional baseline clinical data from a cohort of KOA patients undergoing PRP therapy. Specifically, the dataset included anthropometric parameters, hepatic and renal functional indices, and comprehensive blood biochemical profiles. By systematically comparing the predictive capacity of various machine learning models against observed clinical outcomes, we aimed to develop and validate a high-performance computational framework capable of preemptively predicting early patient response to PRP therapy. Concurrently, we applied advanced interpretability algorithms to explicitly identify the pivotal biomarkers driving the model’s clinical determinations.

## Methods

### Study participants

A retrospective review was performed on patients with knee osteoarthritis undergoing PRP therapy who had complete clinical data and follow-up records. The primary cohort was treated at the Department of Joint Orthopedics, Affiliated Yantai Yuhuangding Hospital of Qingdao University, between January 2010 and June 2024. The Medical Ethics Committee of the hospital approved the study protocol, and informed consent was signed by all patients. Clinical and biochemical measurements recorded prior to PRP injection served as the independent features for modeling. The primary outcome of the study was the 6-month clinical response to PRP therapy. A total of 21 patients were excluded due to loss to follow-up, resulting in a final dataset of 102 patients. The treatment response was defined as a binary variable derived from the Numerical Rating Scale (NRS) pain score: patients demonstrating a reduction in their pain score (Δ*NRS* ≥2) from baseline to the 6-month follow-up were labeled as “responders” (Class 1), whereas those with Δ*NRS* <2 were labeled as “non-responders” (Class 0). This threshold was selected because an approximately 2-point reduction on the 11-point NRS has been shown to represent a clinically important improvement from the patient’s perspective in chronic pain populations (27).

Inclusion Criteria: The inclusion criteria were as follows: • Clinical and radiographic diagnosis of knee osteoarthritis in accordance with the American College of Rheumatology (ACR) criteria; • completion of at least three intra-articular PRP injections; and • complete availability of baseline clinical information, laboratory tests, and 6-month follow-up pain scores.

Exclusion Criteria: The exclusion criteria were as follows: • Co-morbidity with other forms of arthritis, including rheumatoid arthritis or gouty arthritis; • severe knee joint deformity or gross instability; • systemic diseases that could affect PRP quality (e.g., severe hematological diseases or poorly controlled diabetes); and• recent (within 4 weeks) intra-articular injections or oral medications that affect coagulation.

### Data preprocessing and feature engineering

The initial dataset included various raw demographic, anthropometric, and biochemical features. To ensure data quality and robustness for machine learning, a structured preprocessing pipeline was implemented prior to modeling. Missing continuous variables were imputed using the mean, whereas missing categorical variables were imputed using the mode. Ordinal and categorical variables were one-hot encoded where appropriate. We eliminated redundant variables by applying a zero-variance threshold of 0.05 and utilized a correlation threshold of 0.8 to remove highly collinear variables. No external algorithmic feature selection (e.g., Boruta) was applied for variable exclusion; instead, all 33 features that successfully passed these basic filters were preserved as predictors to model the multidimensional clinical profile.

To address the numerical differences in measurement scales, all continuous features subsequently underwent a Yeo–Johnson transformation, followed by standardization using a StandardScaler. Finally, to mitigate the risk of algorithmic bias induced by class imbalance, we applied the SMOTETomek technique to balance the positive and negative sample distributions in the training set. The 33 retained baseline features fell into the following general categories: • Anthropometrics: height, weight, and BMI; • liver function: total protein, globulin, total bilirubin, direct bilirubin, indirect bilirubin, aminotransferases, aspartate aminotransferase (AST)-to-alanine aminotransferase (ALT) ratio, and total bile acids; • kidney function and metabolism: urea-to-creatinine ratio, uric acid, and homocysteine; • enzymes: amylase, phosphatase, creatine kinase, and dehydrogenase;• lipid profile: total cholesterol, triglycerides, and lipoprotein(a); and • clinical/demographics: age, baseline pre-treatment NRS pain rating, sialic acid, and osmotic pressure.

### Model training and optimization

To evaluate the true generalization capability of the algorithms, the balanced, preprocessed dataset was split into a training set (70%) and a test set (30%) using stratified random sampling to maintain the class ratios. We systematically compared the predictive capacity of 17 established classification models, including Gradient Boosting Classifier (GBC), Random Forest, CatBoost, XGBoost, Support Vector Machine (SVM), and Multi-Layer Perceptron (MLP). The model evaluation on the training set was conducted using 10-fold stratified cross-validation. The Gradient Boosting Classifier emerged as the best-performing model. We then employed Optuna to perform hyperparameter tuning using a 10-iteration search optimizing for the area under the curve (AUC). Interestingly, the default hyperparameters (e.g., learning_rate = 0.1, max_depth = 3, n_estimators = 100) outperformed the best Optuna-tuned parameters (mean AUC: 0.68 vs. 0.596). The untuned (default) baseline GBC was thus retained as the final optimal classifier.

### Interpretability analysis and statistical environment

Given the “black-box” nature of ensemble models, we implemented SHapley Additive exPlanations (SHAP) analysis on the final GBC model to calculate the marginal contribution of individual clinical features toward the binary predictions. The entire machine learning pipeline—including data structuring, cross-validation, and visual SHAP analysis—was conducted using Python 3.9 with pandas (v1.5.3), numpy (v1.23.5), scikit-learn (v1.2.2), CatBoost (v1.2), and SHAP (v0.41.0). The final GBC hyperparameters are as stated above; the full pipeline configuration (preprocessing steps and model setup) is provided in the Supplementary File.

## Results

### Feature dimensionality reduction

**Figure 1:**
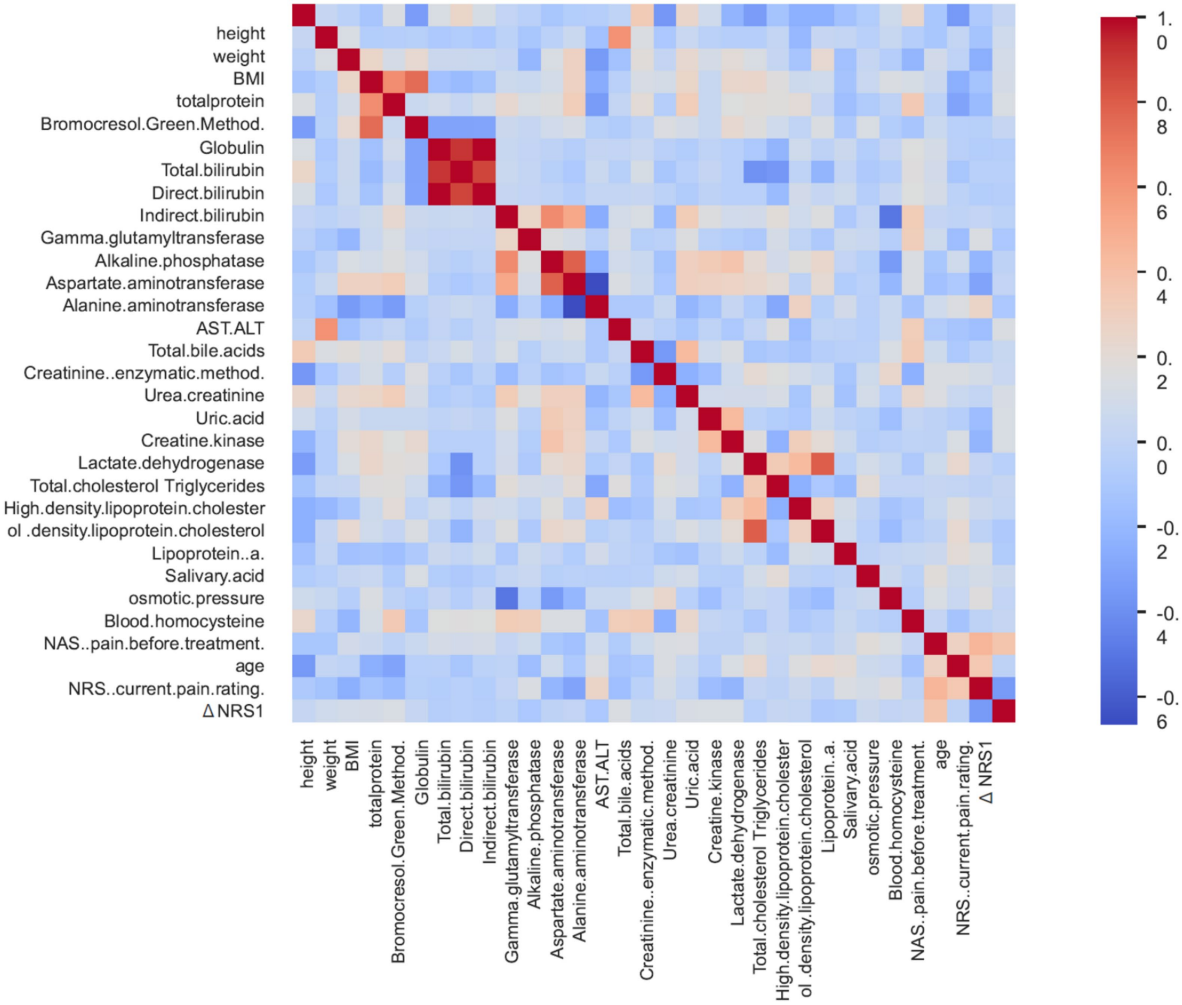
Correlation heatmap.

We performed a correlation analysis to determine the relationships between the 33 variables ultimately included in the Gradient Boosting Classifier modeling (Figure 1). The findings indicated that Pearson’s correlation coefficients among the majority of the variables were low, and no apparent issues of multicollinearity were observed. The baseline disease severity (pre-treatment pain score) was notably positively correlated with age. This finding highlights that the chosen variables maintained a high level of independence, providing a valid database built on distinct, non-overlapping physiological dimensions.

### Model performance

**Figure 2:**
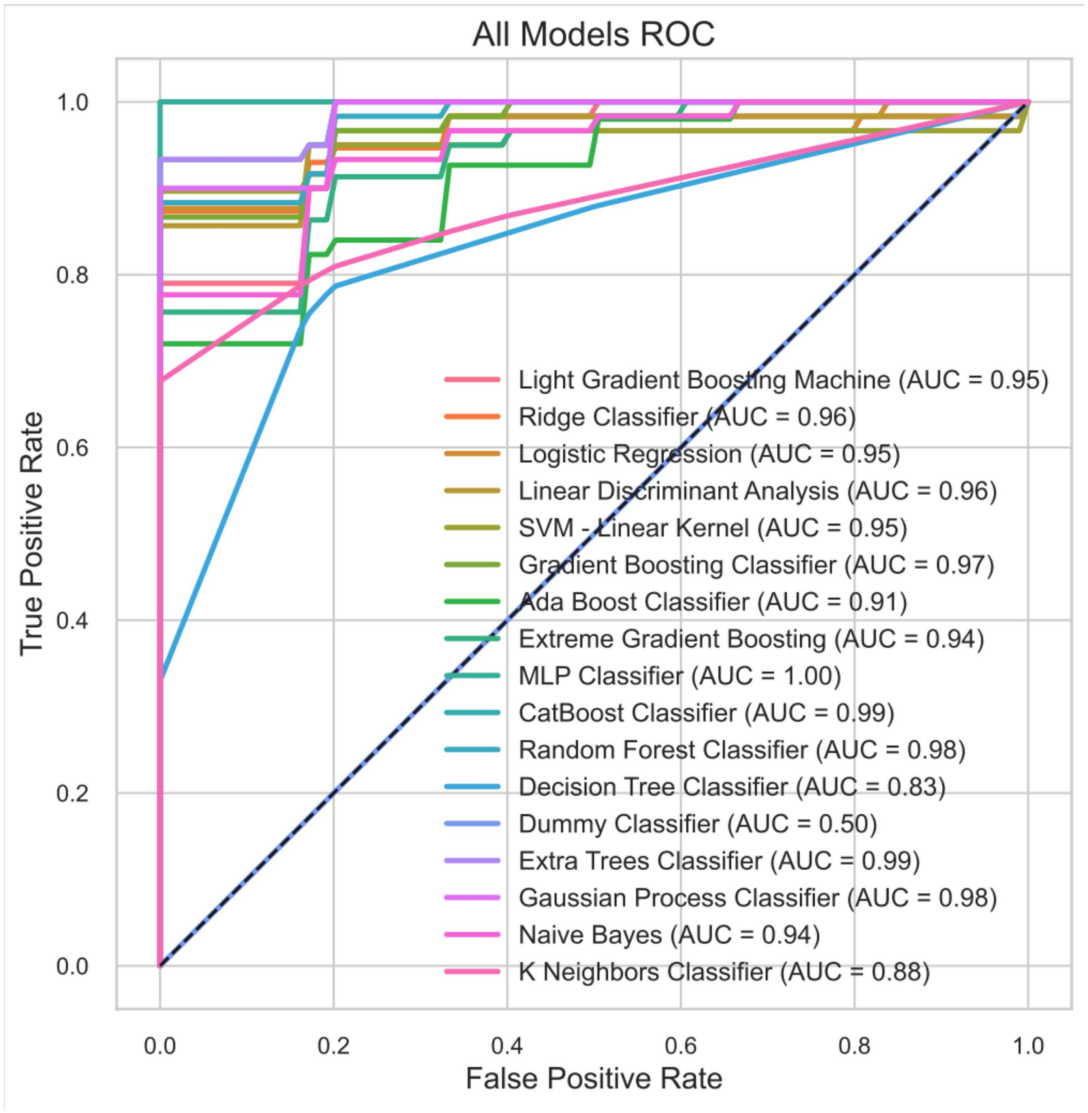
Comparison of receiver operating characteristic (ROC) curves for multi-model classification performance (Train_cv: merged 10-fold CV predictions).

We evaluated candidate classification models using 10-fold cross-validation. Figure 2 illustrates the ROC curves computed from the merged, out-of-fold predictions (Train_cv merged) across the 10 folds for all 17 models evaluated. Measuring discrimination by pooling all cross-validated predictions into a single ROC curve revealed that algorithms such as MLP and Extra Trees achieved extremely high theoretical AUC values (close to 1.0). The Train_cv merged predictions for the Gradient Boosting Classifier achieved an AUC of 0.97.

Table 1 provides the mean cross-validation scores averaged across each validation fold (CV-Val mean). While some algorithms generated superficially higher AUCs in the aggregated curve of Figure 2, the Gradient Boosting Classifier achieved the best balance of actual cross-validation resilience, retaining a kappa coefficient of 0.2514, a log loss of 8.2385, and an F1-score of 0.8516. It outperformed standard boosting and linear algorithms in terms of categorical consistency across varying validation splits. The detailed 10-fold breakdown metrics for the GBC model are provided in Supplementary Table 1. Notably, the apparent mathematical discrepancy between the AUC for GBC in Figure 2 (0.97) and Table 1 (0.68) accurately reflects the difference between scoring predictions aggregated broadly across out-of-fold outputs versus the unweighted averaging of stringent subsets.

**Table 1:** Mean fold validation performance across 10-fold CV.

Model	Accuracy	AUC	Recall	Prec	F1	Kappa	MCC	Log loss	Brier
Light Gradient Boosting Machine	0.7571	0.7167	0.8600	0.8438	0.8475	0.1139	0.1208	8.7535	0.2429
Ridge Classifier	0.7000	0.7133	0.7400	0.8931	0.7919	0.1601	0.2044	10.8131	0.3000
Logistic Regression	0.7286	0.7100	0.7900	0.8764	0.8195	0.1437	0.1698	9.7833	0.2714
Linear Discriminant Analysis	0.7143	0.7033	0.7567	0.8931	0.8052	0.1742	0.2162	10.2982	0.2857
SVM—Linear Kernel	0.7571	0.7000	0.8300	0.8929	0.8434	0.1949	0.2337	8.7535	0.2429
Gradient Boosting Classifier	0.7714	0.6800	0.8433	0.8698	0.8516	0.2514	0.2467	8.2385	0.2286
Ada Boost Classifier	0.8000	0.6567	0.9300	0.8452	0.8828	0.0608	0.0699	7.2087	0.2000
Extreme Gradient Boosting	0.7143	0.6133	0.8600	0.8029	0.8268	0.1275	0.1366	10.2982	0.2857
MLP Classifier	0.7286	0.5933	0.8200	0.8395	0.8147	0.0508	0.0579	9.7833	0.2714
CatBoost Classifier	0.7143	0.5733	0.8567	0.7995	0.8247	0.1371	0.1416	10.2982	0.2857
Random Forest Classifier	0.7857	0.5500	0.9467	0.8190	0.8766	0.0569	0.0592	7.7236	0.2143
Decision Tree Classifier	0.6571	0.5383	0.7267	0.8400	0.7669	0.0775	0.0720	12.3578	0.3429
Dummy Classifier	0.1714	0.5000	0.0000	0.0000	0.0000	0.0000	0.0000	29.8647	0.8286
Extra Trees Classifier	0.8286	0.4317	1.0000	0.8286	0.9051	0.0000	0.0000	6.1789	0.1714
Gaussian Process Classifier	0.5571	0.4267	0.6200	0.8090	0.6850	0.1045	0.1065	15.9622	0.4429
Naive Bayes	0.6857	0.4033	0.7833	0.7971	0.7809	0.0877	0.1016	11.3280	0.3143
K Neighbors Classifier	0.4429	0.3683	0.4267	0.8083	0.5326	0.0686	0.0877	20.0815	0.5571

### Model calibration and final extrapolation

**Figure 3:**
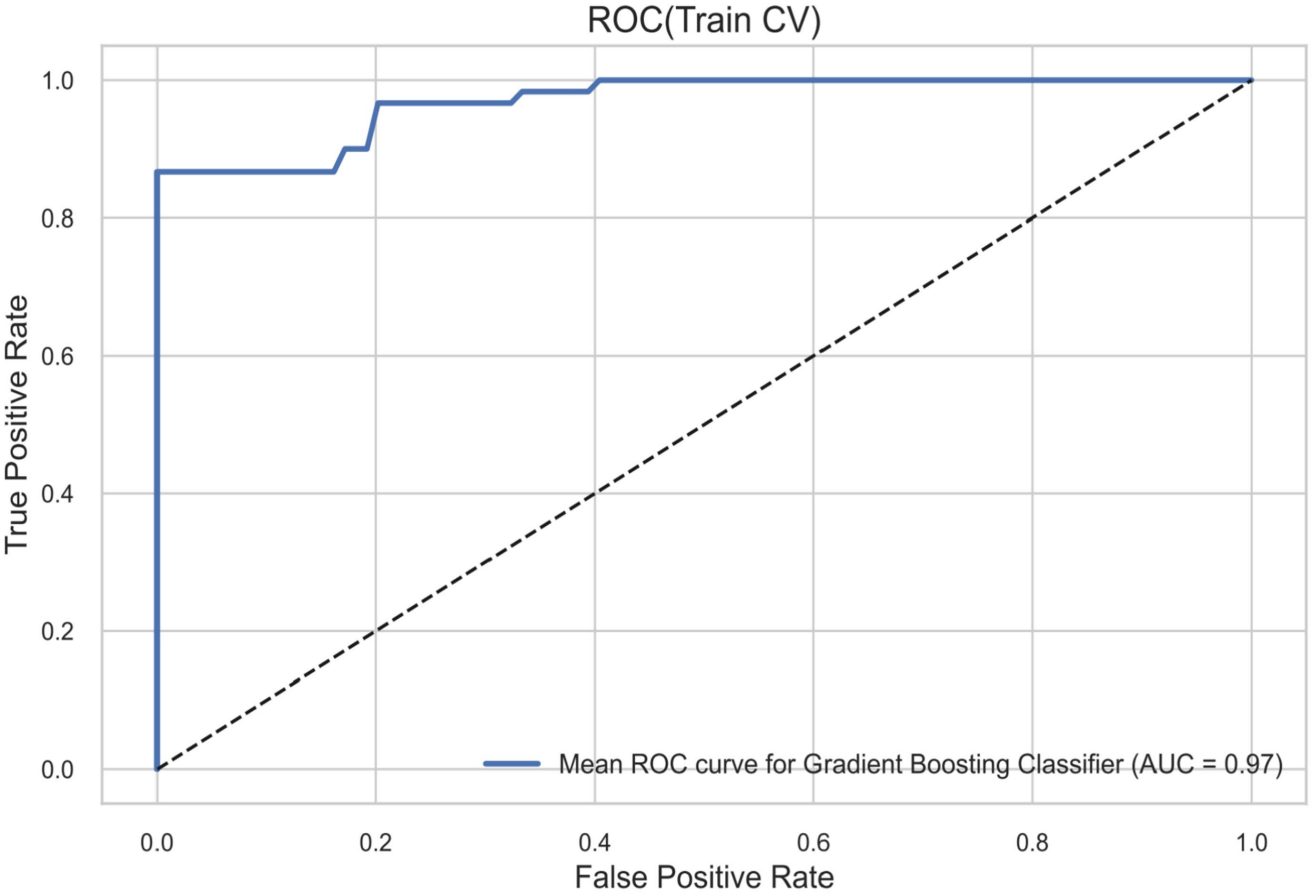
Calibration/ROC based on merged 10-fold CV predictions (Train_cv) on the training set.

**Figure 4:**
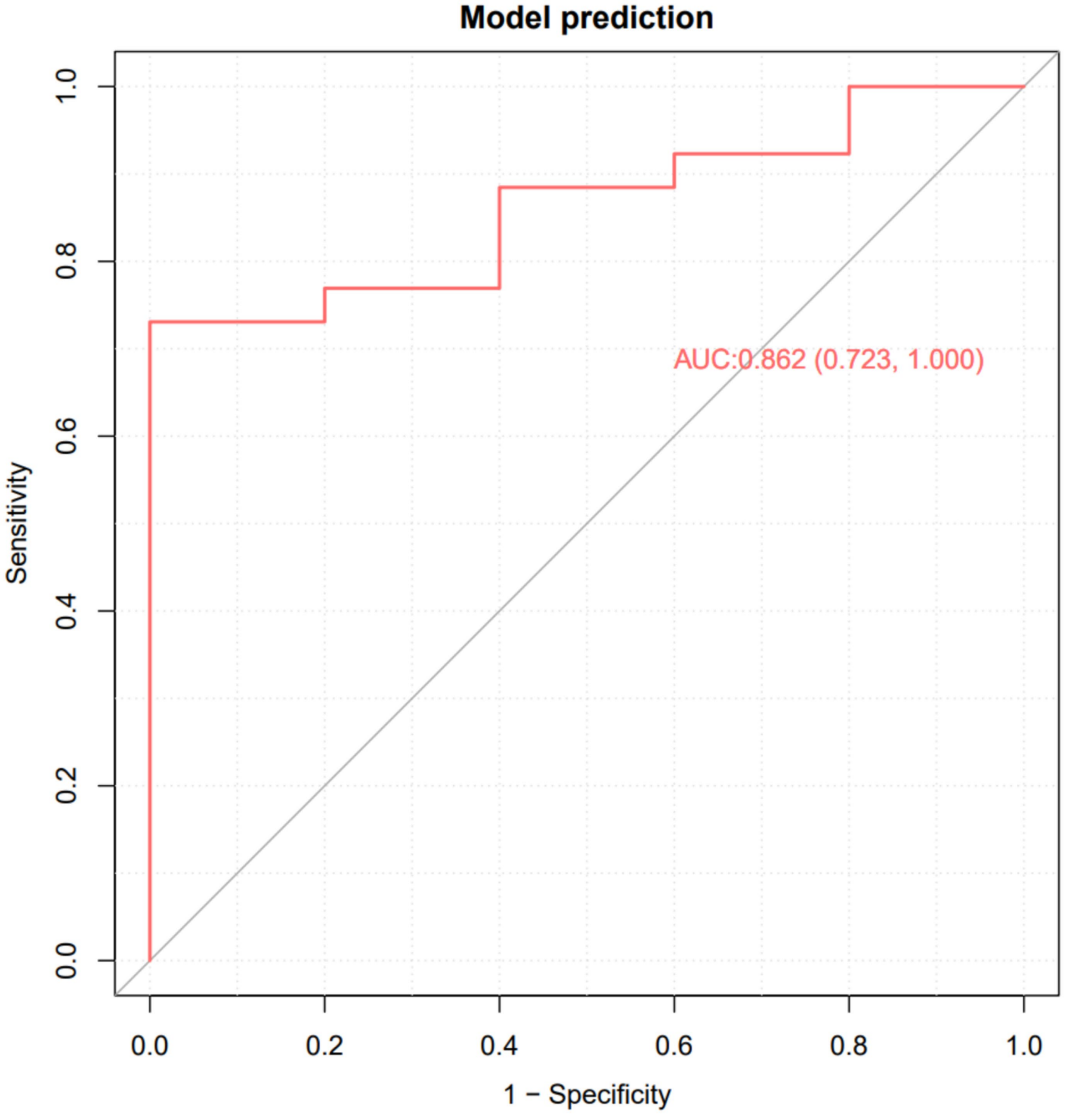
Calibration/ROC on the test set.

To evaluate the performance robustness of the chosen GBC model, we examined its calibration curves representing probability distributions both during cross-validation predictions derived from the training set (Figure 3) and fundamentally against the 30% test set (Figure 4). The calibration index calculated over the test set reflected a highly robust overall accuracy with a test set C-index (calibration AUC) of 0.862. These findings confirm the robustness of the model and its quantitative ability to generalize probabilities appropriately on entirely unseen clinical data, supporting confidence in its use for clinical stratification.

**Figure 5:**
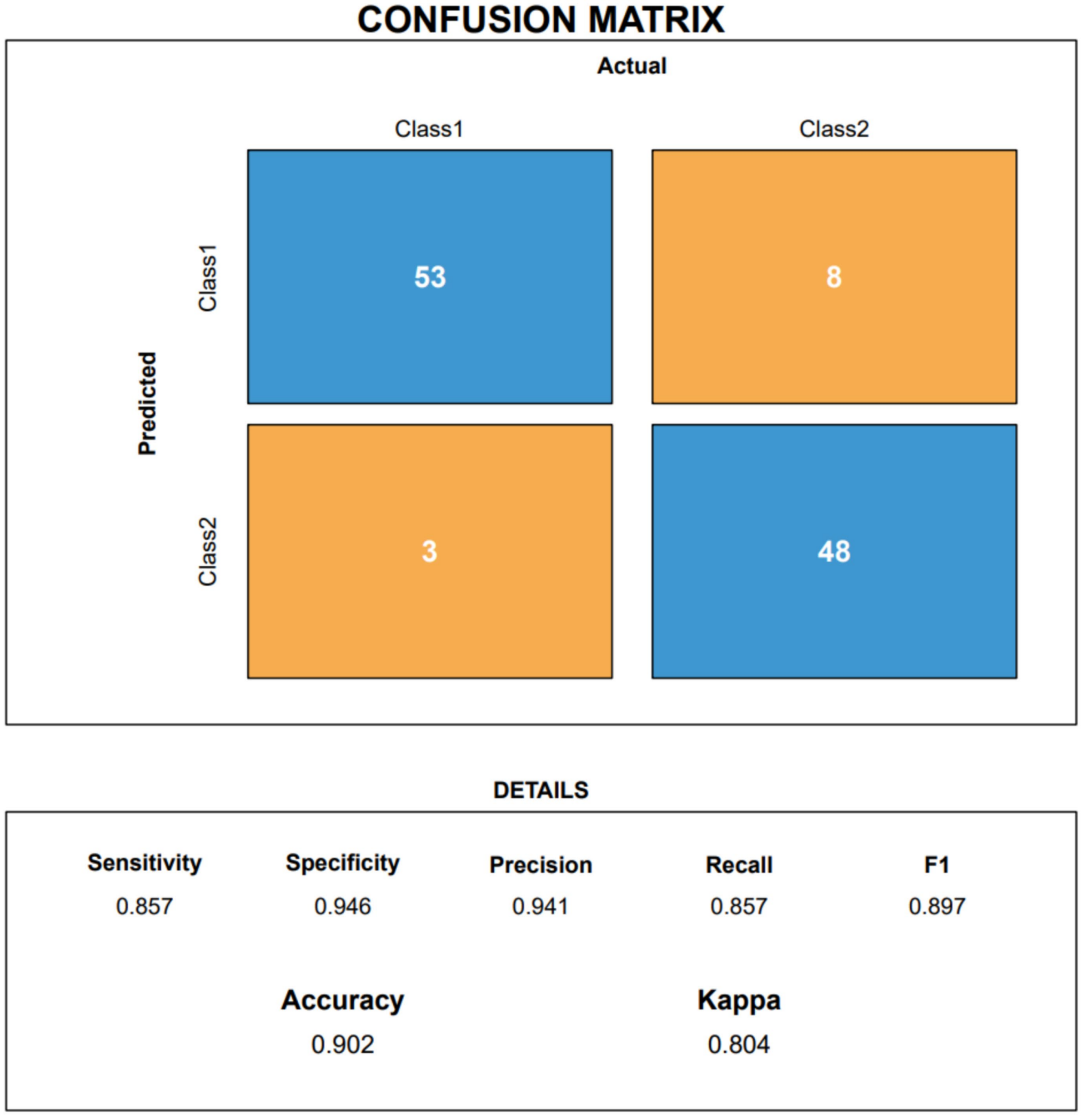
Confusion matrix from merged out-of-fold predictions (Train_cv) on the training set.

**Figure 6:**
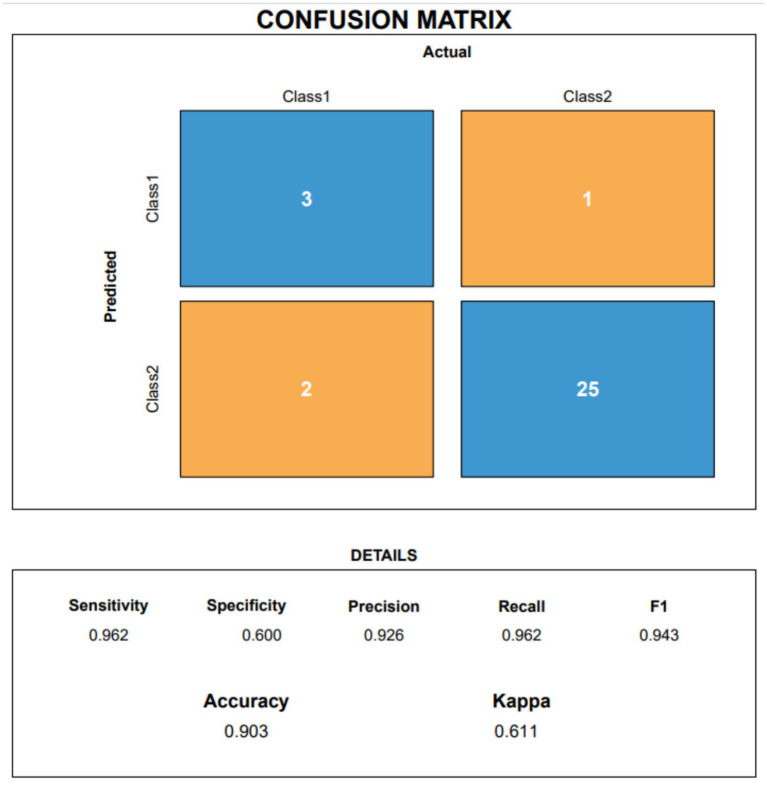
Confusion matrix on the test set.

Figure 5 shows the confusion matrix reconstructed from the merged cross-validation predictions during training, successfully classifying 53 of 56 non-responders and 48 of 56 responders. Most importantly, Figure 6 presents the confusion matrix calculated directly on the unseen test set. The validation demonstrated high general prediction stringency, with an accuracy of 0.903 and a kappa of 0.611. In detecting effective therapies (Class 1), the model correctly identified 25 of 26 true responders (sensitivity = 0.962). While the specificity indicates that the model can occasionally be moderately conservative regarding negative predictions, the F1 score of 0.943 indicates that almost all patients suitable for PRP would be appropriately captured, making the model highly practical for pre-treatment guidance. The relatively lower specificity implies that some false-positive predictions (patients predicted as responders who would not benefit) may occur, which should be considered during shared decision-making to avoid unnecessary treatment when appropriate.

### Model interpretability

**Figure 7:**
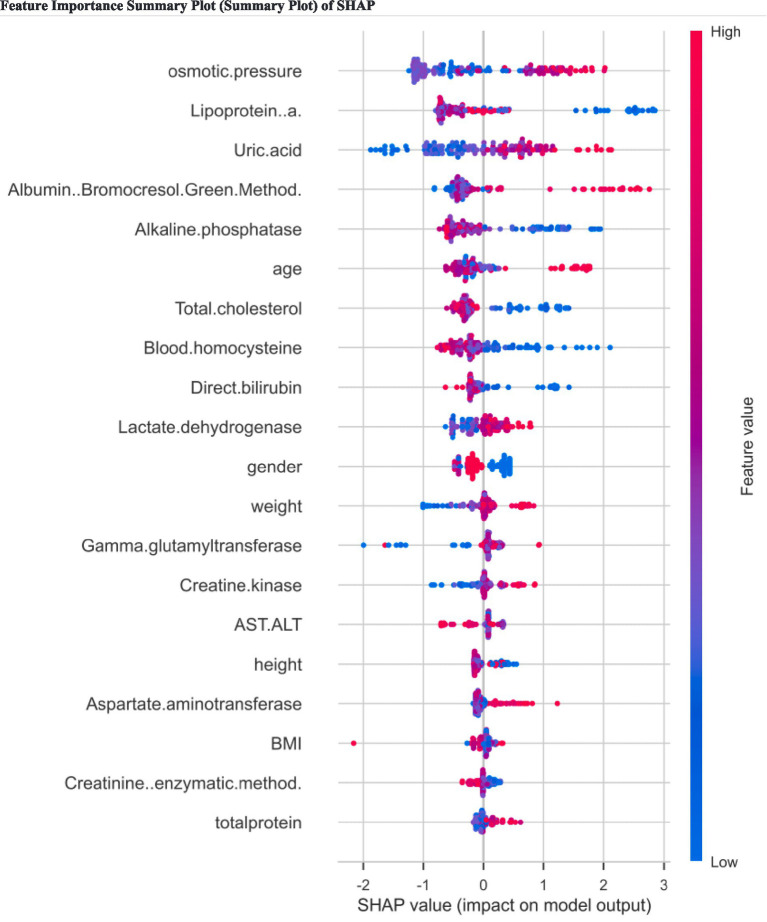
Feature importance summary plot (summary plot) of SHAP.

We plotted the SHAP feature importance summary to investigate how different clinical features were associated with the final prediction outcomes (Figure 7). The plot ranks features in descending order according to their mean absolute SHAP values. Osmotic pressure recorded the largest average absolute impact, making it the most dominant feature associated with predicting therapy outcomes in our model. Lipoprotein(a) and uric acid consistently featured as the second and third most impactful predictors, respectively. Together, these top three biomarkers acted as critical indicators of individual patient profiles. Other clinically relevant factors ranking high included albumin and alkaline phosphatase.

**Figure 8:**
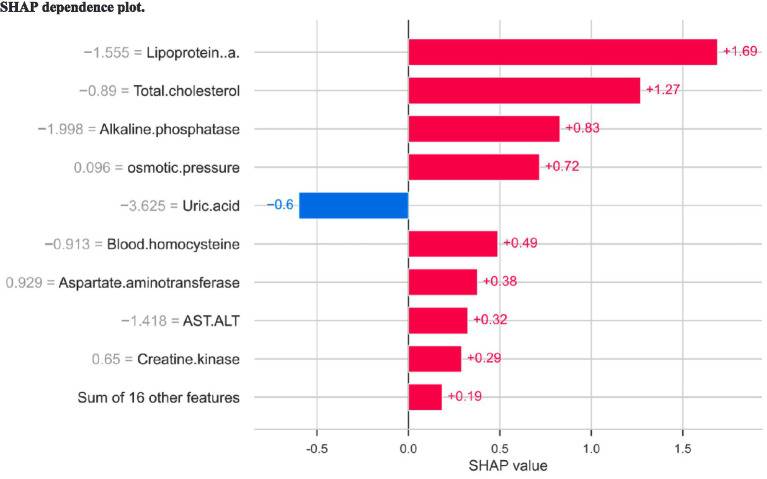
SHAP dependence plot.

The SHAP dependence plot (Figure 8) and local bar plot helped clarify individual patient behavior. Taking a single clinical sample as a representative use case, lipoprotein(a) strongly leaned the predicted probability toward a positive response. For instance, a standardized Lp(a) measurement generated a local marginal contribution (SHAP value) of +1.69. Similarly, baseline total cholesterol contributed positively (+1.27), whereas uric acid contributed a localized negative pressure on the predicted outcome success (SHAP = −0.6).

**Figure 9:**
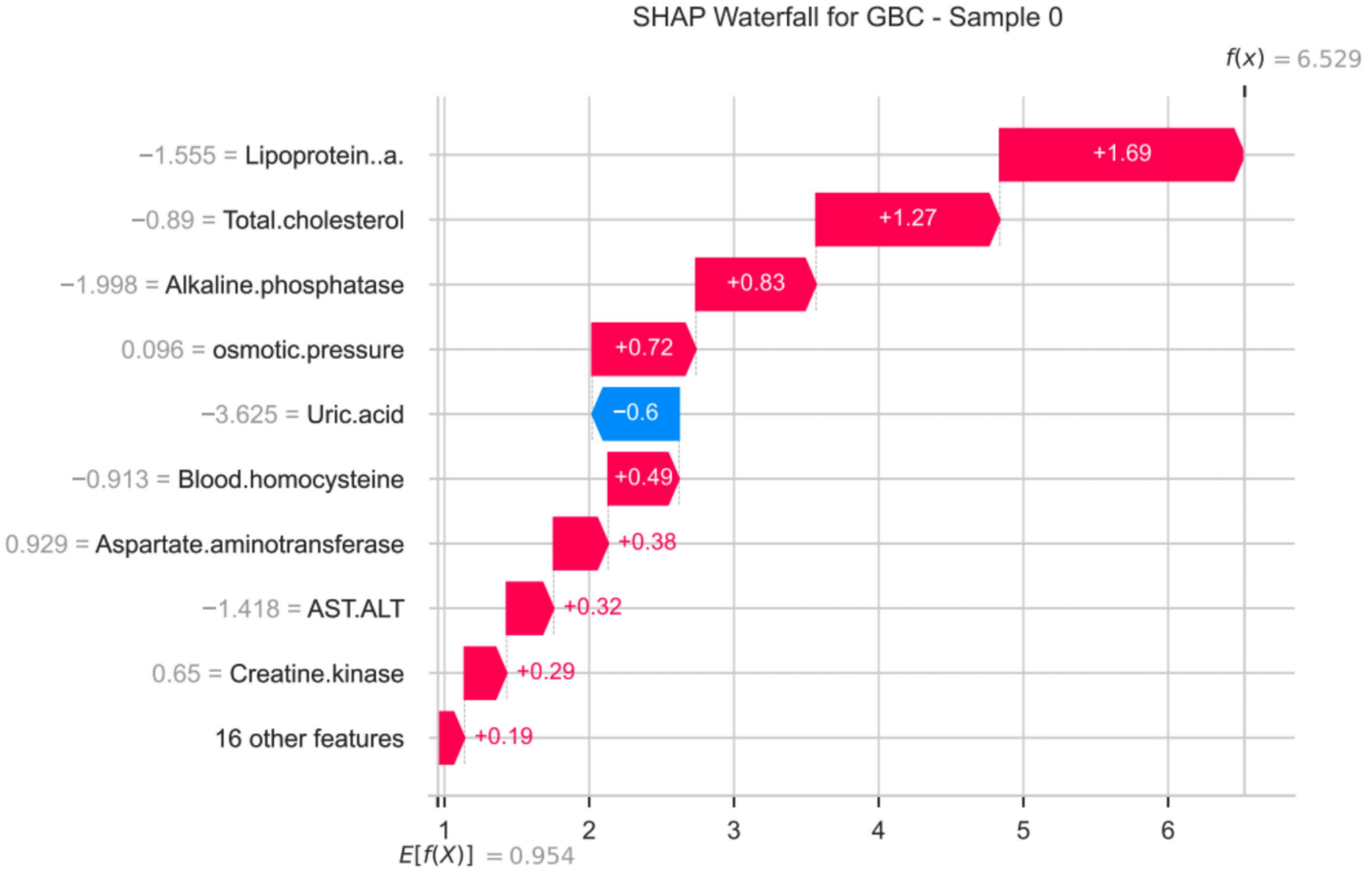
SHAP waterfall plot analysis (for Gradient Boosting Classifier model).

We visualized the predictive logic mapping applied to an individual KOA patient using a SHAP waterfall plot (Figure 9). The baseline expectation (mean *f* (*x*) output log-odds) for the model was 0.954. For this particular sample, lipoprotein(a) (−1.555) augmented the probability of treatment success substantially, generating a net + 1.69 increment upward. Increased baseline alkaline phosphatase and total cholesterol similarly drove the prediction toward clinical efficacy. Conversely, uric acid exerted the strongest dampening effect on predicting a successful response for this patient.

## Discussion

This study successfully constructed and evaluated a machine learning model to predict the clinical response outcome 6 months after platelet-rich plasma (PRP) treatment by integrating multidimensional baseline clinical data, including 33 features such as anthropometrics, liver and kidney function, and basal metabolic and blood biochemical indicators, from 102 patients with knee osteoarthritis (KOA). After comparing 17 mainstream classification algorithms, the machine learning architecture based on Gradient Boosting Classifier (GBC) demonstrated the best and most robust predictive performance. Hyperparameter tuning (Optuna) did not further improve performance; therefore, the default hyperparameter GBC was retained as the final model, which may be because the default regularization already had sufficient constraint and avoided overfitting with the current sample size. The model achieved a classification accuracy of 90.3% and an area under the curve (C-index/AUC) of 0.862 on the test set, with a sensitivity of 96.2%. By introducing *post-hoc* interpretive analysis using the SHAP analysis, we revealed that osmotic pressure, lipoprotein(a), and uric acid are the three core biomarkers that determine the model’s classification and are most strongly associated with PRP efficacy. In this model, these three biomarkers are primarily used for risk stratification and prediction; whether they can serve as interventional therapeutic targets requires further validation in prospective studies.

Comparison with previous PRP efficacy prediction studies provides context for the above results. In a prospective observational study involving 210 knees undergoing PRP, Chopin et al. (28) assessed response at the 7-month time point according to the Outcome Measures in Rheumatology (OMERACT)-Osteoarthritis Research Society International (OARSI) criteria, with a response rate of approximately 43.8%. A multivariate analysis revealed that physical therapy and a heel-to-hip distance of >35 cm were independently associated with poor response, and the response was not significantly associated with radiographic staging (Kellgren-Lawrence). In a retrospective cohort of 517 patients who received three Leukocyte-Poor (LP)-PRP injections, Saita et al. (29) reported that the OMERACT-OARSI response rate was approximately 62.1% at both 6 and 12 months and that worsening of Kellgren-Lawrence (KL) grade (OR: 0.58) was identified as an independent predictor of adverse outcomes, suggesting that radiographic severity may be related to efficacy in some populations. All of the aforementioned studies used traditional multivariate regression analyses, primarily focusing on clinical or radiographic variables, and the endpoint definitions and follow-up time points (7 months vs. 6/12 months) differed from this study (6 months, NRS pain reduction ≥2). Given the extremely high heterogeneity of KOA and significant individual differences in PRP treatment response, such one-dimensional or limited-dimensional predictors yield inconsistent results across different cohorts (28, 29), and traditional linear models struggle to capture the complex, non-linear effects of multi-organ metabolism and systemic physiological state on the local joint microenvironment (15, 17). The novelty of this study lies in its comprehensive baseline serological and metabolic perspective. After basic filtering such as zero variance and collinearity, 33 features were retained.

Through SMOTETomek resampling and multi-model cross-validation, the algorithm autonomously learns within a multi-dimensional feature space, complementing traditional regression methods, such as those of Chopin and Saita, which rely on clinical/imaging indicators. The discovered serological indicators provide a valuable supplement to previous predictive perspectives that primarily focused on imaging and cartilage structure. It should be noted that this study did not directly compare with simple clinical rules or existing predictive models; therefore, the incremental predictive value of the model requires further validation in future studies.

If this predictive model is validated in a multicenter and prospective study, it will have potential value in health economics and clinical applications. While PRP injection therapy has great potential, it carries inherent burdens such as high out-of-pocket costs, acute pain associated with injection, and long treatment duration. The model’s high sensitivity (96.2%) and generalization performance on the hold-out test set suggest that, if validated in independent cohorts and prospective designs, it could be integrated into a clinical decision support (CDS) tool for pre-treatment screening. Before initiating invasive procedures and incurring high medical expenses, physicians can use models to output “high-risk non-response” warnings, combining metabolic indicators from routine blood tests. From an implementation perspective, these key predictive factors are all structured laboratory numerical variables that can be easily mapped to electronic medical records or hospital information systems. This provides a technical foundation for the subsequent development of implementable CDS tools. This screening approach helps manage patient expectations rationally during shared decision-making, providing a reference for considering unicompartmental/total knee arthroplasty (TKA) or other alternative interventions as early as possible for those with poor expected treatment outcomes, thereby moving toward precision medicine for KOA.

The SHAP model emphasizes osmolarity, lipoprotein(a), and uric acid, which are biologically consistent with existing evidence on the pathological progression of KOA and the mechanism of action of PRP and are all supported by independent literature.

Serum uric acid plays the role of a negative predictor of treatment efficacy in the model. Krasnokutsky et al. (30) and Denoble et al. (31) reported that, even in non-gouty KOA patients without a clear tophi attack, high serum uric acid levels can still induce urate microcrystal deposition in synovial fluid, activate NOD-like receptor family pyrin domain containing 3 inflammasomes in synovial innate immune macrophages, and promote cartilage degradation and joint space narrowing. The core effect of PRP depends on the recruitment of stem cells and matrix synthesis by growth factors, such as transforming growth factor-*β* (TGF-β) and platelet-derived growth factor (PDGF), whereas uric acid-induced persistent pyroptosis and the inflammatory cascade may weaken the pro-anabolistic signaling of PRP, leading to “passivation” of the microenvironment, which is consistent with the negative contribution identified by SHAP in this study.

Second, the high influence of osmotic pressure is highly consistent with recent studies on the role of joint microenvironment osmotic pressure in osteoarthritis (OA) pathology. In the journal Advanced Science (2014), Govindaraj et al. (32) demonstrated that the osmotic pressure within the joint microenvironment directly contributes to the development of osteoarthritis (OA) by altering the intracellular molecular crowding of chondrocytes: decreased osmotic pressure in OA joints leads to increased chondrocyte volume and decreased intracellular molecular crowding, thereby making cells more sensitive to pro-inflammatory stimuli and less responsive to anabolic signals; exposing OA chondrocytes to the osmotic pressure at the level of healthy joints can reverse the abovementioned catabolic sensitivity and abnormal glycolysis. This study suggests that serum osmotic pressure may indirectly reflect the state of the whole body fluid and local microenvironment, consistent with the result that osmotic pressure is one of the strongest predictors in this model.

Lipoprotein(a) is closely related to systemic metabolic disorders, inflammatory pathways, and cardiovascular risk. Although there are few studies directly exploring the relationship between Lp(a) and the efficacy of KOA or PRP, the association between metabolic syndrome, type 2 diabetes, and KOA and their impact on the cartilage and synovial microenvironment has been reviewed by Courties et al. (33, 34). Excessively high circulating lipid profiles and their impact on blood rheology and body fluid osmotic environment may alter synovial microcirculation and synovial fluid exchange. As an avascular tissue, cartilage’s nutrition and metabolic waste excretion are highly dependent on the osmotic exchange between synovial fluid and the synovium. Patients with abnormal baseline protein/osmolarity levels may have relatively insufficient autologous biological deficiency in their platelet-rich plasma (PRP), partially explaining the suboptimal therapeutic effect after injection. In summary, the weights of the three biomarkers in the model corroborate existing biological evidence. It should be noted that SHAP reveals the marginal contribution of features to model predictions. While the above mechanistic explanations are consistent with the model results, the causal relationships and whether these biomarkers can serve as interventional targets still require prospective studies for verification.

Although the model performed well on the hold-out test set, this study still has academic limitations, and the extrapolation of conclusions should be approached with caution. First, as a retrospective study, there may be residual confounding biases that were not fully controlled. Second, the sample size was relatively small (*n* = 102), whereas the number of features included in the final model reached 33, resulting in a sample-to-feature ratio of approximately 3:1. Although overfitting was controlled through 10-fold cross-validation and a 30% hold-out test, the multidimensional feature space still posed a certain risk of overfitting at this sample size. Third, the test set sample size was limited (approximately 31 cases), leading to wide confidence intervals for performance metrics, such as sensitivity and specificity, and resulting in estimation uncertainty. Fourth, there was a lack of external validation cohorts from other independent medical institutions. Fifth, the endpoint defined in this study was a 6-month follow-up, with a ≥ 2-point decrease in NRS pain as the response criterion. This differs from the 7-month OMERACT-OARSI used by Chopin et al. (28) and the 6−/12-month OMERACT-OARSI used by Saita et al. (29) in terms of both endpoints and population, potentially affecting direct comparability with previous literature and extrapolation of conclusions. Sixth, the impact of PRP on long-term (e.g., 2–5 years) delay in joint replacement was not assessed.

Furthermore, this study did not directly compare the model with simple clinical rules or existing predictive models, and its incremental predictive value remains to be validated. Future research urgently needs to conduct prospective validation in multicenter, large-sample KOA cohorts and to explore the inclusion of mixed modal data, such as radiomics and inflammatory mediators, to solidify the model’s predictive efficacy over long time periods and across populations.

## Conclusion

Clinical character-based machine learning models show promise in predicting PRP therapy outcomes before administration in this single-center, retrospective cohort. Given the lack of external validation and the limited test set size, generalization of the conclusions should be cautious. With prospective and multicenter validation, such approaches could provide a basis for introducing individualized therapeutic modalities into the practice of knee osteoarthritis management.

## Data Availability

The original contributions presented in the study are included in the article/Supplementary material, further inquiries can be directed to the corresponding author.
