# Supplementary material for: Machine learning predicts clinical response to platelet-rich plasma therapy in knee osteoarthritis
Source: Front Med (Lausanne). 2026 Apr 14;13:1787872. doi: 10.3389/fmed.2026.1787872 (PMC13120951; doi:10.3389/fmed.2026.1787872)
Supplement: Supplementary file 1 [file Supplementary_file_1.DOCX]

# Supplementary File

**Machine learning predicts clinical response to platelet-rich plasma therapy in knee osteoarthritis**

This file provides the full pipeline configuration (preprocessing steps and model setup) for reproducibility.

## 1. Software environment

- Python 3.9
- pandas v1.5.3
- numpy v1.23.5
- scikit-learn v1.2.2
- catboost v1.2
- shap v0.41.0

## 2. Data preprocessing pipeline

| Step | Description |
| --- | --- |
| Missing values (numeric) | SimpleImputer, strategy = mean |
| Missing values (categorical) | SimpleImputer, strategy = most_frequent |
| Low variance | VarianceThreshold, threshold = 0.05 |
| Multicollinearity | RemoveMulticollinearity, threshold = 0.8 |
| Class balancing | FixImbalancer with SMOTETomek (on training set only) |
| Scale / distribution | PowerTransformer, method = yeo-johnson; then StandardScaler |

## 3. Data split and cross-validation

- **Split:** Training set 70%, test set 30%, stratified random sampling (approximately 70 and 31 samples respectively).
- **Cross-validation:** StratifiedKFold, n_splits = 10, applied on the training set only for model comparison and tuning.

## 4. Model comparison and selection

- **Procedure:** compare_models with fold = 10, sort = AUC; 17 classification models evaluated.
- **Best model:** Gradient Boosting Classifier (GBC) by cross-validation resilience (CV-Val mean AUC 0.68, Kappa 0.2514, F1 0.8516).

## 5. Hyperparameter tuning (GBC)

- **Procedure:** tune_model for GBC, search library = Optuna, n_iter = 10, optimize = AUC.
- **Result:** Default GBC (mean AUC 0.68) outperformed the best Optuna-tuned model (mean AUC 0.5967); the default model was retained.

## 6. Final model: Gradient Boosting Classifier (default parameters)

| Parameter | Value |
| --- | --- |
| learning_rate | 0.1 |
| max_depth | 3 |
| n_estimators | 100 |
| random_state | 123 |

(Other parameters: scikit-learn GradientBoostingClassifier defaults.)

## 7. Final set of 33 features

**Anthropometrics:** Height, weight, BMI.

**Liver function:** Total protein, globulin, total bilirubin, direct bilirubin, indirect bilirubin, aminotransferases, AST/ALT ratio, total bile acids.

**Kidney function & metabolism:** Urea/Creatinine ratio, uric acid, homocysteine.

**Enzymes:** Amylase, phosphatase, creatine kinase, dehydrogenase.

**Lipid profile:** Total cholesterol, triglycerides, lipoprotein(a).

**Clinical/Demographics:** Age, baseline pre-treatment NRS pain rating, sialic acid, osmotic pressure.

## 8. Supplementary Table 1

|  |  | Accuracy | AUC | Recall | Prec. | F1 | Кappа | MCC | Log Loss | Brier |
| --- | --- | --- | --- | --- | --- | --- | --- | --- | --- | --- |
| CV-Train | 0 | 0.9841 | 0.6674 | 0.9808 | 0.6746 | 0.9903 | 0.9468 | 0.9482 | 0.5721 | 0.0159 |
| CV-Train | 1 | 0.6995 | 0.7223 | 0.8809 | 0.7997 | 0.8534 | 0.6674 | 0.6876 | 0.8746 | 0.8942 |
| CV-Train | 2 | 0.7775 | 0.7789 | 0.8224 | 0.6697 | 0.7539 | 0.7852 | 0.6673 | 0.6756 | 0.8812 |
| CV-Train | 3 | 0.8123 | 0.7669 | 0.8891 | 0.8882 | 0.8633 | 0.7998 | 0.6379 | 0.6897 | 0.7726 |
| CV-Train | 4 | 08712 | 0.6811 | 0.7659 | 0.6176 | 0.7932, | 0.6045 | 0.7336 | 0.6591, | 0.7123 |
| CV-Train | 5 | 0.6987 | 0.7450 | 0.7021 | 0.6348 | 0.7819 | 0.6675 | 0.7503 | 0.6229 | 0.7761 |
| CV-Train | 6 | 0.6402 | 0.7088 | 0.6894 | 0.6099 | 0.7256 | 0.6734 | 0.7990 | 0.6150 | 0.7607 |
| CV-Train | 7 | 0.6301 | 0.7499 | 0.6518 | 0.7852 | 0.7167 | 0.6923 | 0.7305 | 0.6028 | 0.7700 |
| CV-Train | 8 | 0.6453 | 0.7201 | 0.6830 | 0.7574 | 0.6266 | 0.7915 | 0.6112 | 0.7418 | 0.6609 |
| CV-Train | 9 | 0.7053 | 0.6877 | 0.7382 | 0.7958 | 0.6496 | 0.7145 | 0.7881 | 0.7537 | 0.6385 |
| CV-Val | 0 | 0.7286 | 0.6822 | 0.7833 | 0.75 | 0.6 | 0.2727 | 0.3536 | 0.6889 | 0.5714 |
| CV-Val | 1 | 0.8571 | 0.7001 | 0.8333 | 0.7769 | 0.9091 | 0.5882 | 0.6455 | 0.6491 | 0.1429 |
| CV-Val | 2 | 0.6688 | 0.6339 | 0.7449 | 0.7856 | 0.6899 | 0.6688 | 0.6803 | 0.7602 | 0.7498 |
| CV-Val | 3 | 0.5714 | 0.6667 | 0.6667 | 0.8 | 0.7273 | 0.2353 | 0.2582 | 0.6473 | 0.4286 |
| CV-Val | 4 | 0.8571 | 0.5 | 0.6687 | 0.8571 | 0.9231 | 0.6113 | 0.6428 | 5.1491 | 0.5429 |
| CV-Val | 5 | 0.7143 | 0.5 | 0.8333 | 0.8333 | 0.8333 | 0.1667 | 0.1667 | 0.7982 | 0.2857 |
| CV-Val | 6 | 0.6538 | 0.6238 | 0.7421 | 0.7139 | 0.7266 | 0.7812 | 0.7128 | 0.6413 | 0.7492 |
| CV-Val | 7 | 0.8571 | 0.3333 | 0.6358 | 0.8571 | 0.9231 | 0.6274 | 0.6117 | 5.1491 | 0.1429 |
| CV-Val | 8 | 0.7143 | 0.7 | 0.8 | 0.8 | 0.8 | 0.3 | 0.3 | 5.2982 | 0.2857 |
| CV-Val | 9 | 0.7143 | 0.6 | 0.8 | 0.8 | 0.8 | 0.3 | 0.3 | 4.2982 | 0.2857 |
| CV-Train | Mean | 0.9984 | 0.6671 | 0.9981 | 0.6126 | 0.999 | 0.9947 | 0.9948 | 0.0572 | 0.0016 |
| CV-Train | Std | 0.0048 | 0.5589 | 0.0058 | 0.6671 | 0.0029 | 0.0159 | 0.0155 | 0.1716 | 0.0048 |
| CV-Val | Mean | 0.7714 | 0.68 | 0.8433 | 0.8698 | 0.8516 | 0.2514 | 0.2467 | 0.2385 | 0.2286 |
| CV-Val | Std | 0.1714 | 0.2306 | 0.1585 | 0.0903 | 0.1192 | 0.453 | 0.4698 | 0.4789 | 0.1714 |
| Train | nan | 0.6991 | 0.6423 | 0.7154 | 0.7214 | 0.6324 | 0.7418 | 0.6541 | 0.6892 | 0.6748 |
